# Early‐onset triple‐negative breast cancer in multiracial/ethnic populations: Distinct trends of prevalence of truncation mutations

**DOI:** 10.1002/cam4.2047

**Published:** 2019-03-12

**Authors:** Qian Liu, Song Yao, Hua Zhao, Qiang Hu, Marilyn L. Kwan, Janise M. Roh, Christine B. Ambrosone, Lawrence H. Kushi, Song Liu, Qianqian Zhu

**Affiliations:** ^1^ Department of Biostatistics & Bioinformatics Roswell Park Comprehensive Cancer Center Buffalo New York; ^2^ Department of Cancer Prevention and Control Roswell Park Comprehensive Cancer Center Buffalo New York; ^3^ Department of Epidemiology The University of Texas MD Anderson Cancer Center Houston Texas; ^4^ Division of Research Kaiser Permanente Northern California Oakland California

**Keywords:** cancer screening, early‐onset, health disparity, next‐generation sequencing, triple‐negative breast cancer

## Abstract

Young black women are at higher risk of triple‐negative breast cancer (TNBC); however, a majority of the genetic studies on cancer predisposition were carried out in White populations. The underrepresentation of minority racial/ethnic populations in cancer genetic studies may have led to disproportionate gaps in our knowledge of cancer predisposition genes in these populations. We surveyed the protein‐truncating mutations at the exome‐wide scale and in known breast cancer predisposition genes among 170 patients of multiple racial/ethnic groups with early‐onset (≤age 50) TNBC from two independent cohorts. Black patients, on average, had a higher number of truncating mutations than Whites at the exome‐wide level, but fewer truncating mutations in the panel of known breast cancer genes. White TNBC patients showed a strong enrichment of truncating variants in known breast cancer genes, whereas no such enrichment was found among Black patients. Our findings indicate likely more breast cancer disposition genes yet to be discovered in minority racial/ethnic groups, and the current multigene panels may result in unequal benefits from cancer genetic testing.

## 1 INTRODUCTION

The advent of next‐generation sequencing technology has opened the door to multigene genetic testing, where a panel of cancer genes can be screened simultaneously to evaluate an individual's cancer risk.^1^, ^2^ Despite concerns in interpreting results from multigene testing,^3^ this new version of genetic testing was quickly adopted in clinical setting and resulted in meaningful preventive care for cancer patients and their family members.^4^ The widespread availability of genetic testing provides opportunities to identify individuals with inherited cancer risk and to tailor the preventive actions according to individuals’ genetic makeup. This so‐called “precision prevention” approach was recommended as a national priority by the Cancer Moonshot Blue Ribbon Pane, due to its potential in significantly reducing cancer incidence and mortality.^5^ The Cancer Moonshot Initiative proposed to ramp up efforts in delivering best practice genetic testing, genetic counselling, and preventive care to individuals with high cancer risk across all racial and ethnic groups.^5^

Disparities in healthcare among different ethnic groups have long been noted.^6^ Recently, multiple studies highlighted another disparity, namely the lack of representativeness of minority races/ethnicities in large‐scale genomic data resources and clinical databases, which adds to the health disparities among those populations and may extend the disparities into the upcoming era of “precision prevention” if left unaddressed.^7^, ^8^, ^9^, ^10^ To date, a majority of the genetic studies on cancer predisposition was carried out in populations of European‐descent.^7^, ^11^, ^12^ This lack of diversity in study populations leads to bias in our knowledge of cancer predisposition genes, which is the basis of the gene panels targeted in genetic testing. If known genetic panels were consisted of predisposition genes identified in populations of European‐descent, and lacked predisposition genes in populations of African descent, we could anticipate the genetic screening based on our current knowledge would be more effective in individuals of European ancestry than in individuals of African ancestry, which would further result in bias in cancer prevention.

In this study, we set out to investigate the potential ethnic disparities in genetic testing. We chose to focus on triple‐negative breast cancer (TNBC) because it is the most clinically challenging breast cancer subtype due to lack of targeted therapy and individuals of African ancestry have a markedly higher life‐time risk of TNBC than other ethnicities.^13^, ^14^, ^15^, ^16^, ^17^, ^20^

## 2 MATERIALS AND METHODS

### 2.1 Study population

DNA samples were available for sequencing from 126 early‐onset (age <= 50) triple‐ negative female breast cancer patients (TNBC) from four racial/ethnic groups. These included 48 Whites, 40 Blacks, 25 Hispanics, and 13 Asians. Those samples were obtained from the Pathways Study, a prospective cohort study of women diagnosed with breast cancer in Kaiser Permanente Northern California (n = 106)^18^ and from the Data Bank and BioRepository (DBBR)^19^ at Roswell Park Cancer Institute (n = 15). The study was approved by Institutional Review Boards (IRB) at Roswell Park Cancer Institute and Kaiser Permanente Northern California.

### 2.2 Next‐generation sequencing, variant calling and quality assessment

Exome capture was performed on 126 samples using Agilent SureSelect Human Exon v5 capture kit from the genomic DNA isolated from each individual. The captured DNA was sequenced using Illumina HiSeq2500 to generate 100‐bp paired‐end reads. Raw sequence reads were aligned to the Human Reference Genome (NCBI Build 37), using the Burrows‐Wheeler Aligner (BWA).^34^ After removing PCR duplicates using Picard,^35^ the GATK software^36^ was used for local realignment, base quality recalibration, and variant calling of single nucleotide variants (SNVs) and small insertions and deletions (indels). In the variant calling step, variants were first called in each sample separately, and then joint genotyping analysis was performed across all samples to generate analysis‐ready variants. After sample quality assessment using SeqSQC,^37^ samples failed sample quality check which resulted in a total of 116 samples being retained for the downstream analysis.

### 2.3 Variant filtering

Bi‐allelic variants with read depth <3 were considered missing and any variants with missing rate >0.1 were removed. Inherited mutations known to increase cancer risk are usually rare in the general population, therefore it is necessary to exclude common variants from the analysis. However, due to concerns of the lack of diversity in public genomic data resources and clinical databases,^7^, ^8^, ^9^, ^10^ the filtering strategy based on allele frequency in public data sources may introduce bias toward non‐European populations, especially for missense variants with uncertain pathogenicity. Thus, only protein‐truncating variants (splice site, frameshift indels, stop gain and stop loss) were evaluated, and a relatively loose minor allele frequency cutoff of 2% was used to exclude common variants based on data from the 1000 Genomes Project^38^, ^39^ (ALL population, 2015 August release), the Exome Sequencing Project (ESP)^40^, ^41^ (ESP6500siv2 all), and the Exome Aggregation Consortium (ExAC)^42^ (exac03nontcga). Long insertions and deletions (>20 bp), variants with read depth <15, and variants in tandem repeat or segmental duplications^43^ regions were also filtered out. ANNOVAR^44^ was used to facilitate these variant filtering steps.

### 2.4 Breast cancer gene panel

Our panel included *BARD1* plus 18 genes shared between the 114 known cancer predisposition genes^21^ and two commonly used commercial gene panels Myriad myRisk panel^26^ and Invitae Breast and Gyn Cancers panel.^4^ The 18 shared genes include *ATM*,* BRCA1*,* BRCA2*,* BRIP1*,* CDH1*,* CHEK2*,* MLH1*,* MSH2*,* MSH6*,* NBN*,* PALB2*,* PMS2*,* PTEN*,* RAD51C*,* RAD51D*,* STK11*,* TP53*, and *MUTYH*. *BARD1* was included in the gene panel as it was reported to be frequently mutated in TNBC.^45^

### 2.5 Statistical methods

Poisson test was used to test the hypothesis that the observed number of mutation events is different from random in each population. Mutation rate was estimated within each ethnic group as $\lambda_{i}=\sum_{j=1}^{n_{i}}M_{i,j}/\sum_{j=1}^{n_{i}}L_{i,j}$, where *M*_*i,j*_ is the number of all truncating mutations (with read depth ≥ 15, mapping quality > 20, base quality > 13 and ignoring anomalous read pairs) observed in the *j*th individual of the *i*th ethnic group and *L*_*i,j*_ is the accumulated positions within the entire capture regions that have read depth ≥ 15 (mapping quality > 20, base quality > 13 and ignoring anomalous read pairs) in the corresponding individual. “poisson.test” in R was used to test the hypothesis that the observed number of mutation events is different from the random in each population where $x=\sum_{j=1}^{n_{i}}m_{i,j},r=\lambda_{i},\;\text{and}\;T=\sum_{j=1}^{n_{i}}l_{i,j}.m_{i,j}$ is the number of truncating mutations observed in the gene panel and *l*_*i,j*_ is the number of positions within the gene panel that fall in the capture regions and have read depth ≥ 15 (mapping quality > 20, base quality > 13 and ignoring anomalous read pairs) in the corresponding individual. A two‐sided *P* < 0.05 (Type I error rate) is considered statistically significant.

Mann‐Whitney *U* test was used to test whether the number of mutation events in Blacks is different from other populations. Fisher exact test was used to test the difference in mutation rates between Whites and other racial/ethnic groups.

### 2.6 Variant calling in TNBC patients from TCGA

WES data obtained from samples of 62 early onset (age <= 50) TNBC patients (all female, 39 Whites, 22 Blacks, and one Asian) were downloaded from The Cancer Genome Atlas (TCGA).^46^ The TCGA WES data were performed using customized versions of the Agilent SureSelect All Exome v2.0 kit or Nimblegen SeqCap EZ Human Exome v2.0.^47^ As a result, only the intersections between the capture regions of the two kits were included in the analysis. Variant calling, quality assessment, and variant filtering were performed as described above, and a total of 55 samples (32 Whites, 22 Blacks, and one Asian) passed quality assessment. The analysis was focused on 54 samples from the white and the black ethnicity groups.

### 2.7 Variant filtering in ExAC

VCF of variant sites from 53 105 non‐TCGA samples was downloaded from the ExAC website. Variants from four populations of interest (27 173 Whites (Non‐Finnish European); 4533 Blacks (African); 5608 Hispanic (Latino); 3933 Asian (East Asian)) that passed GATK quality filter were further filtered as described above. Because the ExAC data came from a variety of sequencing projects using different exome capture kits, to ensure common capture regions across all samples, we restricted our analysis on variants within the Consensus Coding Sequence (CCDS).

## 3 RESULTS

**Figure 1:**
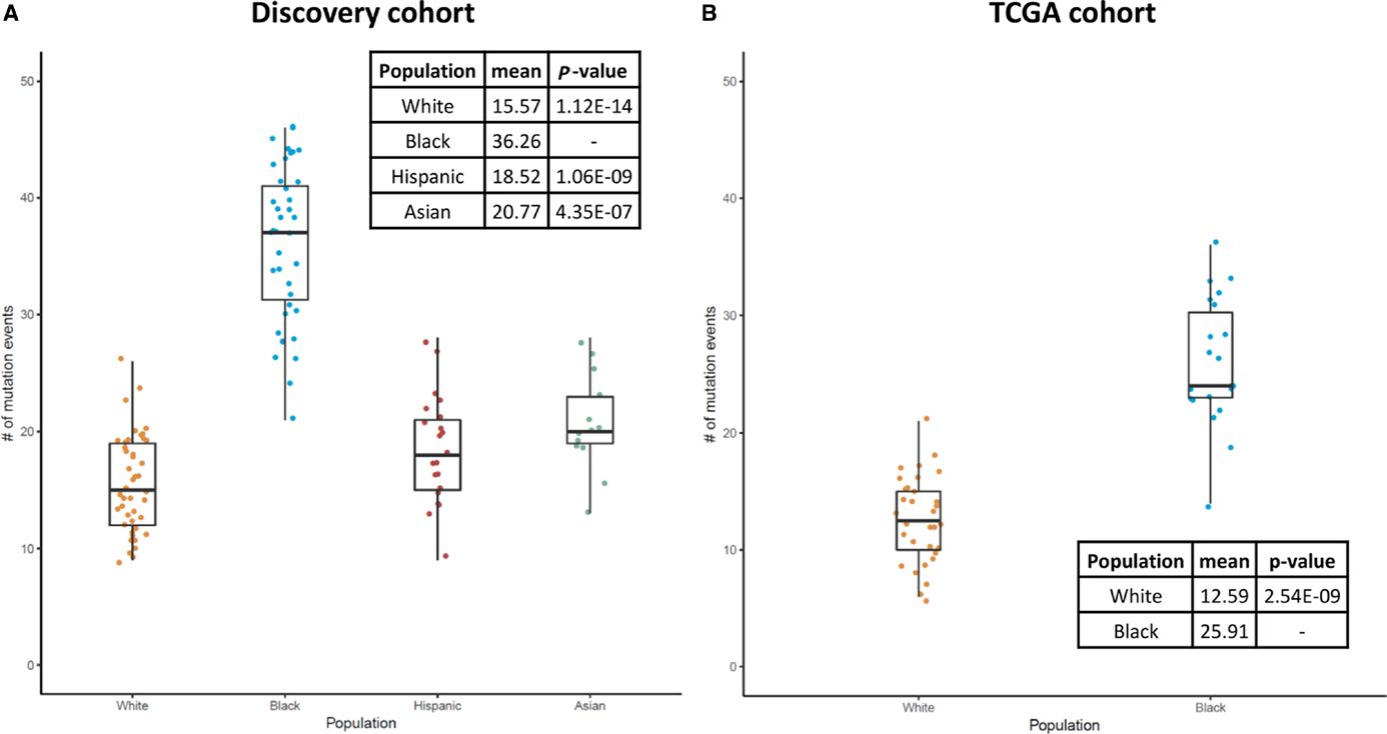
The distribution of the number of mutation events per individual exome‐wide across different populations in our discovery cohort (A) and the TCGA cohort (B). Inset: the mean number of mutation events in each population and the two‐sided *P‐*value from Mann–Whitney *U* test by comparing each population with Blacks

**Figure 2:**
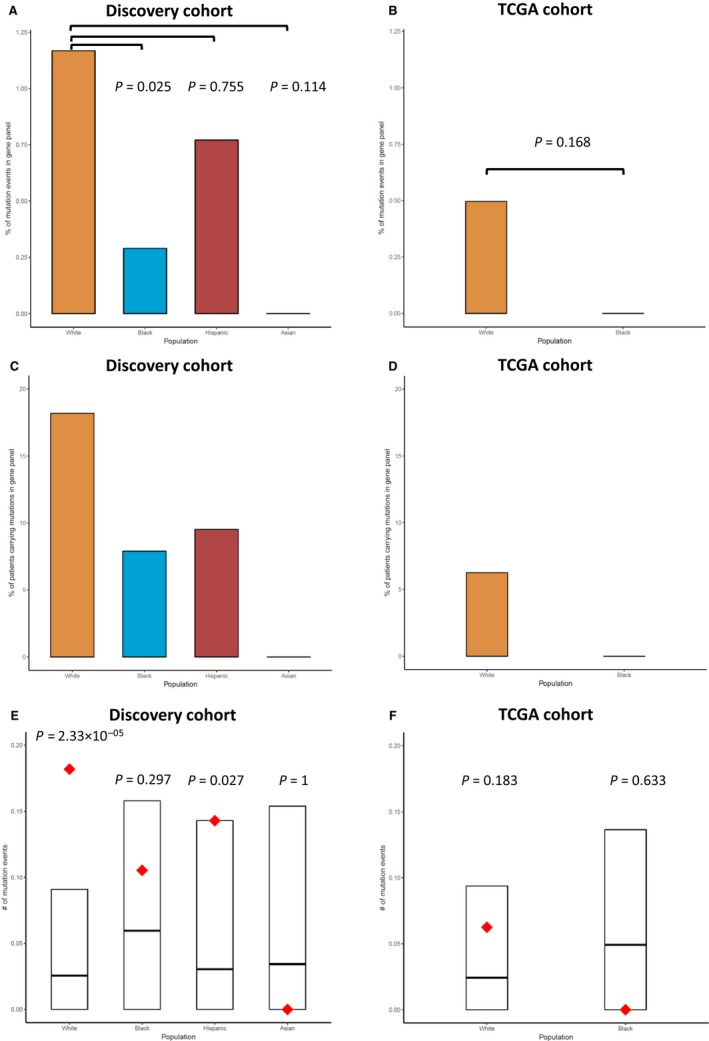
Mutation burden in the Breast Cancer Panel across different populations. (A, B) The percentage of mutation events in the gene panel within each population and the Fisher exact test *P* values (two‐sided) between Whites and other racial/ethnic groups. (C, D) The corresponding percentage of mutation carriers within each population. (E, F) The normalized number of mutation events in the gene panel per individual across different populations. The number of mutation events was normalized by the sample size of each population when plotting. The observed number of mutation events in the breast cancer‐specific gene panel is in red. The 95% confidence interval of random expectation is indicated by the black box with the center line corresponding to the estimated mean. Poisson test was used to test the hypothesis that the observed number of mutation events is different from random in each population. The corresponding two‐sided *P*‐values were labeled on the plots. The samples included in the analysis either came from our discovery cohort (A, C, E) or the TCGA cohort (B, D, F)

Because data are scarce on the carrier status of risk variants among breast cancer patients from minority populations, we performed whole‐exome sequencing (WES) in a multi‐ethnic cohort of 116 patients who were diagnosed with early‐onset (≤age 50) triple‐negative breast cancer (TNBC) (Table S1). At the whole‐exome level, Blacks, on average, had the highest number of truncating mutations among the four racial/ethnic groups in the discovery cohort (Figure 1A). The higher mutation burden in Black cases than in Whites was confirmed in a validation cohort of 54 early‐onset TNBC patients from The Cancer Genome Atlas project (TCGA) (32 Whites and 22 Blacks) (Figure 1B). When focused on a panel of 19 known breast cancer genes (Breast Cancer Panel, Table S2), strikingly, the trend was reversed such that Whites had a higher mutation rate in the breast cancer genes than other racial/ethnic groups (Fisher exact test two‐sided *P *=* *0.025, for Whites vs. Blacks) (Figure 2A). The same trend persisted in TCGA cohort (Figure 2B) and in a meta‐analysis combining both cohorts (Fisher exact test meta‐*P *=* *0.028). Consistently, a higher percentage of Whites carried truncating mutations in the Breast Cancer Panel than other populations (Figure 2C‐D). When the gene panel was expanded to include 114 known pan‐cancer genes^19^ (Pan‐Cancer Panel, Table S2), the same trends were observed (Figure S1).

Because all cases were early‐onset TNBC, we hypothesized that they carried more damaging mutations in breast cancer predisposition genes than random expectation based on population‐specific background mutation rate across the exome. When the observed number of protein‐truncating variants in the Breast Cancer Panel was examined, Whites and Hispanics had significantly more mutations than random (Poisson test two‐sided *P *=* *2.33 × 10^−5^ and 0.027 respectively); however, in Blacks and Asians, no such enrichment was observed (Figure 2E). Although TCGA cohort did not show a significantly higher observed number of mutations than random in Whites (Figure 2F), likely due to limited sample size, meta‐analysis confirmed a significant enrichment of truncating mutations in the gene panel than random expectation in Whites (Poisson test meta‐*P *=* *5.71 × 10^−5^).

**Figure 3:**
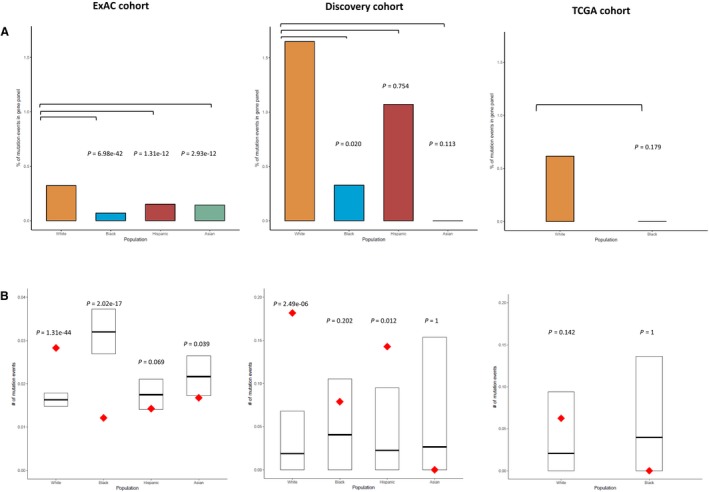
Mutation burden in the Breast Cancer Panel in ExAC cohort, the discovery cohort, and TCGA cohort. To be consistent with ExAC cohort, analyses were restricted to variants inside CCDS. (A) The percentage of mutation events in the gene panel within each population in the three study cohorts. Fisher exact test *P* values (two‐sided) between Whites and other racial/ethnic groups are also included. (B) The normalized number of mutation events in the gene panel per individual across different populations in the three study cohorts. The number of mutation events was normalized by the sample size of each population when plotting. The observed number of mutation events in the breast cancer‐specific gene panel is in red. The 95% confidence interval of random expectation is indicated by the black box with the center line corresponding to the estimated mean. The Poisson test was used to test the hypothesis that the observed number of mutation events is different from random in each population. The corresponding two‐sided *P*‐values were labeled on the plots

In the general populations from the Exome Aggregation Consortium (ExAC), the same trends were observed. Whites had a significantly higher mutation rate in the Breast Cancer Panel genes (Fisher exact test two‐sided *P *=* *6.98 × 10^−42^, for Whites vs. Blacks) (Figure 3A). Consistently, we observed a significant enrichment of truncating mutations for whites in the Breast Cancer Panel than expected based on population‐specific baseline exome‐wide mutation rate (Poisson test two‐sided *P *=* *1.31 × 10^−44^); whereas Blacks carried significantly less mutations than random expectation (Poisson test two‐sided *P *=* *2.02 × 10^−17^) (Figure 3B). It should be noted that the observed mutation rates in almost all general populations were expectedly much lower (>6‐fold) than the rates observed in our breast cancer cohort (Figure 3B), confirming that the genes in the panel were indeed predictive of breast cancer risk across populations. The only exception was in Asians, which is probably due to low sample size of this population in our cohort.

## 4 DISCUSSION

An assumption for the use of the multi‐gene panels in genetic testing across racial/ethnic groups is that the testing is equally beneficial to all groups. Our study now calls into question of this assumption. Although some previous studies reported comparable prevalence of *BRCA1/2* mutations across racial/ethnic groups,^21^, ^22^ the different background mutation rates were not properly considered and the analysis was limited to only two genes. In our study, Black women harbored the largest pool of germline protein‐truncating mutations, but the trend was reversed when the scope was limited to known breast cancer genes, such that Black patients carried fewer truncating mutations in the known cancer genes than White patients. The fact that we did not find an enrichment of truncating variants in known breast cancer genes among Black women is particularly striking, as they are considered at high risk of early‐onset TNBC whereas the signal is unmistakably strong among White women who are at relatively lower risk.

There are two possible explanations to our findings. First, the lower mutation burden in known breast cancer genes could indicate that TNBC in Black women has a relatively minor genetic underpinning than that in White women. This is consistent with a lower percent of Black breast cancer patients who had a positive family history of breast cancer when compared to Whites.^23^ If this speculation is true, it would also imply that non‐genetic risk factors play a very important role in TNBC etiology among Blacks, presumably more prominent than that among Whites.

However, the above speculation would be in contradiction to earlier age onset of TNBC in Black women and inconsistent with our finding that Blacks have a significantly higher germline mutation burden across the exome. The larger pool of rare germline mutations in Blacks are expected, as it is well established that the genomes of Blacks are more diverse, characterized by a larger number of unique variants.^24^ Therefore, it is possible that the fewer mutations in known breast cancer genes observed among Blacks in our study is due to the incomplete knowledge of predisposition genes in that group, as previous studies were predominantly Whites. This suggests that there might be a considerable lack of information on cancer predisposition genes in Blacks.

The prevalence of White patients with TNBC in our study carrying truncating mutations in breast cancer disposition genes (18.2%) is similar to previous studies which invariably relied on multigene panels.^25^, ^26^, ^27^, ^28^ Nevertheless, the mutation prevalence in Black patients with TNBC in our study (7.9%) is lower than other studies in Blacks, which ranged from 12.4% in those with breast cancer at or before age 50^29^ and 14.7% in Nigerians diagnosed mostly at a late stage,^30^ to 14.6%^28^ and 25%^31^ in those with TNBC. Moreover, two previous studies reported similar mutation prevalence across race/ethnicity for women with breast cancer^27^ as well as for those with TNBC.^28^ The discrepancy might be attributed to different definitions used to classify mutations of interest. The confidence level in the pathogenicity of missense mutations vary considerably, and the classification of this class of mutations as “pathogenic” in the current databases (eg, ClinVar) is particularly dubious for non‐European populations, as demonstrated in the study by Manrai and colleagues.^10^ Because the goal of our study is to compare germline mutations across multiple racial/ethnic groups, we felt important to use a stringent definition for pathogenicity to avoid potential biases due to race/ethnicity. We focused more narrowly on truncating mutations with unequivocal evidence of damaging functionality.^32^ As a result, we should emphasize that our findings apply only to truncating mutations but not to more broadly defined pathogenic mutations for which missense mutations are included. Second, the discrepancy in mutation frequency may reflect variations in the underlying populations where the patients were drawn. Notably, the mutation frequency in the study by Pal et al^29^ and Churpek et al^31^ differed by almost twofold; while in our analysis of TCGA data, the mutation rate is lower than our discovery patient population applying the same definition of truncating mutations. These variations probably also reflect the fact that early‐onset TNBC is rare and each study on itself is based on relatively small sample size. Data from Blacks and other minorities are even more sparse. With more data emerging from many large ongoing sequencing initiatives, we may be able to get to a more reliable estimate of the mutation frequency across race/ethnicity.

In recent years, it has been increasingly recognized that extensive ethnic disparities exist in cancer genomic studies, including the lack of representation of minority groups in genomic studies^23^ and the bias of more misclassification of pathogenic variants and genetic misdiagnosis among minority groups.^33^ Our findings provide observation for an additional facet of the disparities, ie, there may be more undiscovered cancer predisposition genes or more environmental risks in Black populations than we had previously suspected. These could potentially limit the ascertainment power of genetic testing based on current multigene panels for Black women and other minorities as mutations in these genes are not as prevalent in minorities as in Whites. Our study, along with others, calls for future endeavors to diversify ethnic groups participating in genomic studies.

## 5 CONCLUSIONS

We conducted a whole‐exome sequencing study in a multi‐ethnic cohort of women diagnosed with triple‐negative breast cancer at or before age 50. At the exome‐wide level, AA cases on average had the highest number of truncating mutations in comparison to EA, Asian and Hispanic cases. However, the trend was reversed when focusing on mutations in the known breast cancer genes, such that AA cases carried fewer mutations. Analysis of data from TCGA confirmed our findings. Our findings suggest that more breast cancer genes may yet to be discovered in AA and other minority populations and call for future endeavors to diversify ethnic groups participating in genomic studies.

## CONFLICTS OF INTEREST

The authors declare no conflict of interest.

## Supporting information


** **

** **

** **
