# Supplementary figures and images for: Early‐onset triple‐negative breast cancer in multiracial/ethnic populations: Distinct trends of prevalence of truncation mutations
Source: Cancer Med. 2019 Mar 12;8(4):1845–53. doi: 10.1002/cam4.2047 (PMC6488149; doi:10.1002/cam4.2047)

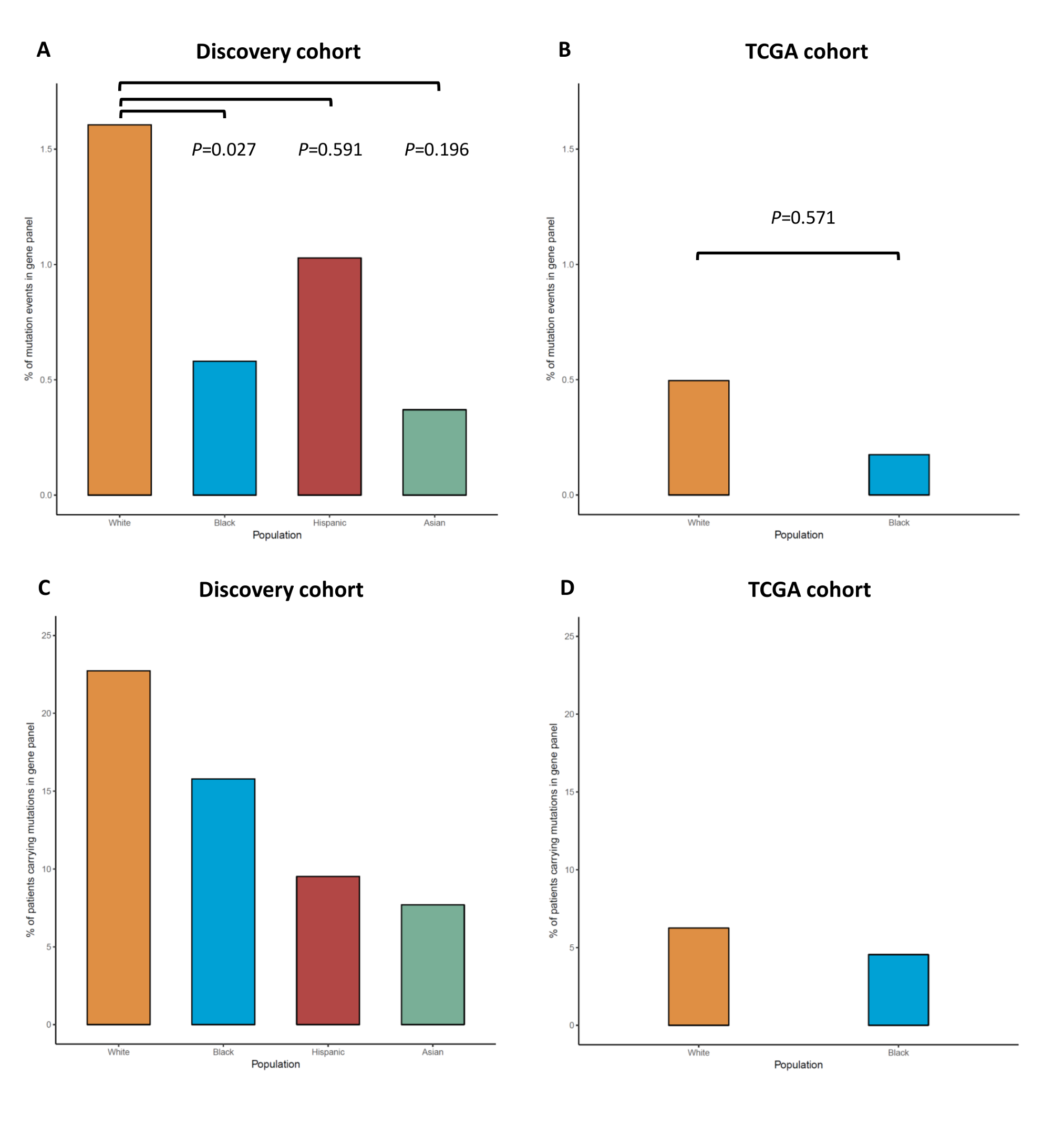

Supplement: Supplementary file 1 [file CAM4-8-1845-s001.tif]
